# Retraction Note: Tumor microenvironment and immune system preservation in early-stage breast cancer: routes for early recurrence after mastectomy and treatment for lobular and ductal forms of disease

**DOI:** 10.1186/s12865-024-00663-7

**Published:** 2024-10-17

**Authors:** Hassan A. Saad, Azza Baz, Mohamed Riad, Mohamed E. Eraky, Ahmed El-Taher, Mohamed I. Farid, Khaled Sharaf, Huda E. M. Said, Lotfy A. Ibrahim

**Affiliations:** 1 Surgical Department, Faculty of Medicine, Zagazig University, Zagazig City, 44661 Egypt; 2 Surgical Department, Alahrar Teaching Hospital, Zagazig University, Zagazig City, 55971 Egypt; 3 Clinical Pathology Department, Faculty of Medicine, Zagazig University, Zagazig City, 55971 Egypt; 4 Surgical Department, AlAzhar University, Nasr City, Cairo 55888 Egypt

**Retraction Note: BMC Immunology (2024) 25:9**

10.1186/s12865-023-00591-y

The Editor has retracted this article because it contains material that substantially overlaps with [1, 2]. The corresponding author Hassan A. Saad has stated that all authors agree with this retraction.
